# 

**Published:** 1888-01

**Authors:** 


### CONTENTS:

ARTICLE I.—Southern Dental Association ........................................ 385
" II.—A New Method of Treating Teeth with Diseased Pulps. By Wilhelm Herbst ........................................ 399
" III.—Improved Artificial Teeth. By Dr. D. Genese ............ 402
" IV.—Odontomes. By J. Bland Sutton, F. R. C. S ................ 405
" V.—On the Combination of Tin and Gold as a Filling Material for the Teeth. By W. D. Miller, M. D., Ph. D., &c.. 418

### EDITORIAL, ETC.

Bridge-Work and Tooth Crown Patents ........................................ 426

### OBITUARY.

Hugh McGinnis Grant, D. D. S ........................................ 428

### MONTHLY SUMMARY.

Two Cases of Epulis ........................................ 430

Lodgment of a Tooth Plate in the Gullet for Fifteen Months ............ 431

A Case of Somewhat Remarkable Character ........................ 432

